# Elevated serum neutrophil-lymphocyte ratio is associated with worse long-term survival in patients with HBV-related intrahepatic cholangiocarcinoma undergoing resection

**DOI:** 10.3389/fonc.2022.1012246

**Published:** 2022-10-17

**Authors:** Jianwei Liu, Yong Xia, Feng Xue, Caixia Lu, Jie Wang, Chunyan Wang, Yeye Wu, Shilei Bai, Feng Shen, Kui Wang

**Affiliations:** ^1^ Department of Hepatic Surgery II, the Eastern Hepatobiliary Surgery Hospital, Naval Medical University, Shanghai, China; ^2^ Department of Hepatic Surgery IV, the Eastern Hepatobiliary Surgery Hospital, Naval Medical University, Shanghai, China

**Keywords:** intrahepatic cholangiocarcinoma, inflammation markers, nlr, liver resection, survival

## Abstract

**Background:**

This study aimed to examine the influence of serum inflammatory marker levels on long-term outcomes after liver resection in patients with intrahepatic cholangiocarcinoma (ICC).

**Methods:**

Data from 1189 consecutive ICC patients who underwent liver resection were reviewed. The serum neutrophil-lymphocyte ratio (NLR), platelet-lymphocyte ratio (PLR), and prognostic nutritional index (PNI) were measured before surgery. Overall survival (OS) and tumour recurrence were analysed using the Kaplan–Meier method and compared using the log-rank test. Independent risk factors for OS and tumour recurrence were analysed using the Cox hazard regression model.

**Results:**

We identified elevated serum NLR (≥ 2.15) as an independent risk factor for both OS and tumour recurrence (hazard ratio [HR]: 1.327, 95% confidence interval [CI]: 1.105-1.593; HR: 1.274, 95% CI: 1.074-1.510) among the three inflammatory markers assessed. Elevated NLR was associated with higher carbohydrate antigen 19-9 (CA19-9) and carcinoembryonic antigen (CEA) levels, larger tumour size, multiple tumours, lymph node metastasis, vascular invasion, and more advanced tumour node metastasis (TNM) stage (III/IV). Subgroup analysis showed that elevated NLR was an independent risk factor for OS and tumour recurrence in patients with hepatitis B virus (HBV) infection compared with patients without HBV infection (HR: 1.347, 95% CI: 1.073-1.690; HR: 1.386, 95% CI: 1.112-1.726).

**Conclusions:**

Elevated serum NLR was associated with worse prognosis among ICC patients who underwent liver resection, especially in patients with HBV infection.

## Introduction

Intrahepatic cholangiocarcinoma (ICC) accounts for 10-15% of primary hepatic malignancies, with an incidence inferior to that of hepatocellular carcinoma (HCC) (1). The incidence of ICC varies greatly among regions and continues to increase worldwide (2–4). Studies have demonstrated that multiple risk factors are associated with the development of ICC (2). Hepatitis B virus (HBV) infection is an important risk factor for the disease, particularly in Asia and other ICC high-incidence regions (5, 6). Although liver resection is the only established treatment to achieve a possible cure for ICC, its effectiveness is still far from unsatisfactory due to the high recurrence rate. The 5-year postoperative survival rate has been reported to be 20–35% (7–9).

Serum inflammatory markers can reflect the inflammatory condition in the body and can be detected by serum or obtained by simple calculation. Common inflammatory markers are the neutrophil-to-lymphocyte ratio (NLR), platelet-to-lymphocyte ratio (PLR), and prognostic nutritional index (PNI). Previous studies have demonstrated that serum inflammatory markers, such as NLR, may be associated with prognosis in patients with multiple types of cancer (10–18). In addition, an elevated NLR is reportedly a prognostic risk factor for ICC patients with various risk factors (19, 20). It has been well recognised that ICC with different causative risk factors have obvious biological heterogeneity in tumour origin, gene mutations, oncological signalling pathways, and tumour microenvironment (2). However, the prognostic role of inflammatory markers in ICC patients with different causative factors has not been reported.

This study aimed to examine the impact of serum inflammatory markers, including NLR, PNI, and PLR, on the long-term prognosis after liver resection for ICC. Furthermore, such effects were tested in patients with or without HBV infection.

## Patients and methods

### Patients

Data from consecutive patients who underwent liver resection for ICC between 2012 and 2016 at the Eastern Hepatobiliary Surgery Hospital (EHBH) were reviewed. Patients were included if they had no history of preoperative anti-cancer treatment, including transarterial chemoembolization (TACE), percutaneous radiofrequency ablation, or percutaneous ethanol injection, had no history of other malignancies, and underwent R0 resection for histopathologically proven ICC (21). Patients were excluded if they had mixed types of primary liver cancer, perioperative mortality, or incomplete clinical data. The study was approved by the ethics committee of EHBH. Informed consent was obtained from all patients prior to surgery for data to be used for research.

### Preoperative examination and liver resection

Detailed history enquiries and complete physical examinations were performed for all patients. The routine preoperative examination included liver and renal function tests, HBV and hepatitis C virus (HCV) antigen/antibody tests, HBV DNA level, and tumour biomarkers, including a-fetoprotein (AFP), carbohydrate antigen 19-9 (CA19-9), and carcinoembryonic antigen (CEA). Imaging studies included chest X-ray or non-contrast computed tomography (CT), abdominal ultrasound, contrast-enhanced CT, and/or magnetic resonance imaging (MRI) of the abdomen. Positron emission tomography (PET) was performed in patients with clinical or radiological suspicion of intra- or extrahepatic metastasis. A preoperative diagnosis of ICC was mainly based on a combination of the above examinations.

The criteria for partial hepatectomy for ICC were as follows: (a) patients aged < 80 years with a World Health Organization (WHO) performance status of 0–1 before surgery; (b) solitary or multiple intrahepatic tumours were evaluated to be technically resectable; (c) the estimated volume of future liver remnant was > 30% in normal liver and > 40% in cirrhotic liver; (d) liver function of Child-Pugh grade A and Child-Pugh grade B and the score is 7; and (e) no evidence of extrahepatic distant metastasis. Patients with suspected perihepatic lymph-node metastasis on preoperative CT scan or PET were also considered for operation, if these nodes could be removed safely, as judged preoperatively by experienced surgeons. Anatomical liver resection is the preferred operation if the tumour is within a segment, sector, or hemiliver. Non-anatomical resection was performed for the peripherally situated lesions. Routine dissection of the lymph nodes in the hepatoduodenal ligament and retropancreatic and/or para-aortic lymph nodes was performed for either preoperatively or intraoperatively diagnosed ICC (22). Intrahepatic nodules and direct invasion of adjacent structures were also resected intraoperatively whenever technically possible. Hepaticojejunostomy was performed for patients with tumours involving the primary and secondary bile ducts.

Histopathological analysis of the resected specimens was independently performed by three pathologists who arrived at a consensus by discussion if there was any controversy (22). Pathological features, such as tumour diameter, tumour number, tumour capsule, surgical margin, vascular invasion, node metastasis, and cirrhosis were documented, and the degree of cell differentiation was determined.

### Follow-up and endpoints

Patients were observed once every two months in the first two years and every 3-6 months thereafter. Tests for liver function, CA19-9, and CEA levels, and abdominal ultrasound were performed at each follow-up visit. Contrast-enhanced CT or MRI was performed every six months or earlier if tumour recurrence was clinically suspected. Tumour recurrence or metastasis was diagnosed based on evidences from two radiological imaging studies, with or without elevation of serum tumour markers, and was aggressively treated using a multimodal approach as previously reported (23).

Overall survival (OS) and time to recurrence (TTR) were used as primary endpoints. OS was calculated from the date of operation to the date of patient death or the last follow-up. TTR was defined as the interval from the date of operation to the date of recurrence or metastasis.

### Statistical analysis

Variables are expressed as ratios or medians (inter quartile range, IQR). Continuous variables were compared using the paired *t*-test or Mann–Whitney U test, as appropriate. Categorical variables were compared using the chi-squared test or Fisher’s exact test. Tumour recurrence and OS curves were depicted using the Kaplan–Meier method and compared using the log-rank test. Multivariate analyses of OS and recurrence were performed using the Cox proportional hazard model. The NLR was calculated as the absolute neutrophil count divided by the absolute lymphocyte count. The PLR was calculated as the absolute platelet count divided by the absolute lymphocyte count. The PNI was calculated as 10 × serum albumin value (g/dl) + 0.005 × peripheral lymphocyte count (per mm^3^). The cut-off values for NLR, PLR, and PNI were determined using a minimum *p-*value approach for the entire cohort of patients (24). Hazard ratio (HR) and 95% confidence interval (CI) represent relative risks. Statistical analyses were performed using the IBM SPSS Statistics for Windows software (version 26.0; IBM Corp., Armonk, NY, USA). All reported *p-*values were two-sided, and statistical significance was set at p < 0.05.

## Results

### Patient characteristics

A total of 1189 patients who met our inclusion criteria were enrolled in this study. As shown in **Table 1**, among these patients, the median (IQR) levels of serum AFP, CEA, and CA19-9 were 3.5 (2.2–8.3) µg/L, 2.7 (1.6–5.2) µg/L, and 43.9 (16.0–229.6) U/ml, respectively. The median tumour diameter was 5.7 (4.0–8.0) cm; 174 patients (13.1%) had hepatolithiasis, 21 patients (1.8%) had HCV infection, 345 patients (29.0%) had multiple tumours, 215 patients (18.1%) had lymph-node metastasis, and 246 patients (20.7%) had vascular invasion.

**Table 1:** Baseline clinicopathological data.

Variable	Number (%)/median (IQR)			*P-value*
	Total (n = 1189)	NLR < 2.15 (n = 439)	NLR ≥ 2.15 (n = 750)	
**Age**, years	55.0 (47.0-62.0)	55.0 (48.0-62.0)	55.0 (47.0-62.0)	0.788
**Sex**,				
**Female**	397 (33.4%)	140 (31.9%)	257 (34.3%)	0.402
**Male**	1792 (66.6%)	299 (68.1%)	493 (65.7%)	
**Hepatolithiasis**,				
**NO**	1015 (86.9%)	374 (85.2%)	641 (85.5%)	0.898
**Yes**	174 (13.1%)	65 (14.8%)	109 (14.5%)	
**HBsAg**				
**Negative**	615 (51.7%)	189 (43.1%)	426 (56.8%)	<0.001
**Positive**	574 (48.3%)	250 (56.9%)	324 (43.2%)	
**HBeAg**				
**Negative**	1064 (89.5%)	383 (87.2%)	681 (90.8%)	0.055
**Positive**	125 (10.5%)	56 (12.8%)	69 (9.2%)	
**HBcAb**				
**Negative**	409 (34.4%)	124 (28.2%)	285 (38.0%)	0.001
**Positive**	780 (65.6%)	315 (71.8%)	465 (62.0%)	
**Anti-HCV**				
**Negative**	1168 (98.2%)	429 (97.7%)	739(98.5%)	0.305
**Positive**	21 (1.8%)	10 (2.3%)	11 (1.5%)	
**TBIL**, µmol/L	12.4 (9.4-16.5)	12.3 (9.5-15.7)	12.5 (9.4-17.2)	0.240
**ALB**, g/L	42.3 (39.6-44.8)	42.6 (40.4-44.9)	42.1 (39.1-44.7)	0.009
**ALT**, U/L	26.2 (17.3-42.7)	27.3 (18.6-41.6)	26.0 (16.9-43.8)	0.151
**PT,** seconds	11.6 (11.1-12.2)	11.6 (11.0-12.2)	11.6 (11.1-12.2)	0.326
**AFP**, µg/L	3.5 (2.2-8.0)	3.5 (2.3-7.6)	3.5 (2.2-8.6)	0.951
**CEA**, µg/L	2.7 (1.6-5.2)	2.4 (1.6-4.5)	3.0 (1.6-6.4)	0.003
**CA 19-9**, U/mL	43.9 (16.0-229.6)	33.5 (14.6-111.7)	57.2 (17.0-365.3)	<0.001
**NLR**	2.5 (1.8-3.6)	1.7 (1.4-1.9)	3.3 (2.7-4.2)	<0.001
**PLR**	119.2 (89.1-160.2)	94.0 (73.4-117.8)	140.1 (106.3-189.2)	<0.001
**PNI**	50.2 (46.8-53.6)	52.2 (48.9-55.7)	49.2 (45.7-52.2)	<0.001
**Operation time**, hours	2.0 (1.5-2.8)	2.0 (1.5-2.5)	2.0 (1.5-3.0)	0.003
**Hilar clamping**, minutes	15.0 (6.0-22.0)	15.0 (7.0-21.0)	16.0 (5.8-22.3)	0.188
**Gross type**				
**Mass-forming**	1164 (97.9%)	423 (96.4%)	741 (98.8%)	0.005
**No mass-forming**	25 (2.1%)	16 (3.6%)	9 (1.2%)	
**Cirrhosis**				
**No**	943 (79.3%)	312 (71.1%)	631 (84.1%)	<0.001
**Yes**	246 (20.7%)	127 (28.9%)	119 (15.9%)	
**Tumour diameter**, cm	5.7 (4.0-8.0)	4.6 (3.5-6.4)	6.4 (4.6-8.7)	<0.001
**Tumour number**				
**Solitary**	844 (71.0%)	339 (77.2%)	505 (67.3%)	<0.001
**Multiple**	345 (29.0%)	100 (22.8%)	245 (32.7%)	
**Adjacent organs invasion**				
**No**	1106 (93.0%)	415 (94.5%)	691 (92.1%)	0.117
**Yes**	83 (7.0%)	24 (5.5%)	59 (7.9%)	
**Lymph node metastasis**				
**No**	974 (81.9%)	382 (87.0%)	592 (78.9%)	<0.001
**Yes**	215 (18.1%)	57 (13.0%)	158 (21.1%)	
**Vascular invasion**				
**No**	943 (79.3%)	362 (82.5%)	581 (77.5%)	0.040
**Yes**	246 (20.7%)	77 (17.5%)	169 (22.5%)	
**Differentiation**				
**Poor**	54 (4.5%)	23 (5.2%)	31 (4.1%)	0.377
**Moderate/Well**	1135 (95.5%)	416 (94.8%)	719 (95.9%)	
**TNM**				
**I/II**	926 (77.9%)	365 (82.9%)	561 (74.9%)	0.001
**III/IV**	263 (22.1%)	75 (17.1%)	188 (25.1%)	

IQR, interquartile range; HBsAg, hepatitis B surface antigen; HBeAg, hepatitis Be Antigen; HBcAb, hepatitis B core antibody; HCV, hepatitis C virus; TBIL, total bilirubin; ALB, Albumin; ALT, alanine aminotransferase; PT, prothrombin time; AFP, a-fetoprotein; CEA, carcinoembryonic antigen; CA 19-9, carbohydrate antigen 19-9; NLR, neutrophil to lymphocyte ratio; PLR, Platelet-Lymphocyte Ratio; PNI, prognostic nutritional index; TNM, tumour node metastasis.

Of these patients, 788 were HBsAg- or HBcAb-positive, while the other 401 did not have HBV infection (hepatolithiasis-related or other aetiology-related). The median (IQR) values of the NLR, PLR, and PNI were 2.5 (1.8–3.6), 119.2 (89.1–160.2), and 50.2 (46.8–53.6), respectively. The cut-off values for NLR, PLR, and PNI were 2.15, 141, and 46.5, respectively, for further prognostic analysis, which were determined based on a minimum p-value approach for all 1189 patients (**Supplemental Figure 1**). Based on the cut-off values, 750 and 439 patients presented with NLR ^high^ and NLR ^low^, respectively; 417 and 772 patients presented with PLR ^high^ and PLR ^low^, respectively; and 913 and 276 patients presented with PNI ^high^ and PNI ^low^, respectively.

### OS and tumour recurrence in the entire group

The median follow-up was 32.3 (range, 1.1 to 109.0) months. Among the 1189 ICC patients, the 1-, 3-, and 5-year OS rates were 65.9%, 39.0%, and 28.8%, respectively, and the corresponding tumour recurrence rates were 43.7%, 65.6%, and 75.2%, respectively.

**Figure 1:**
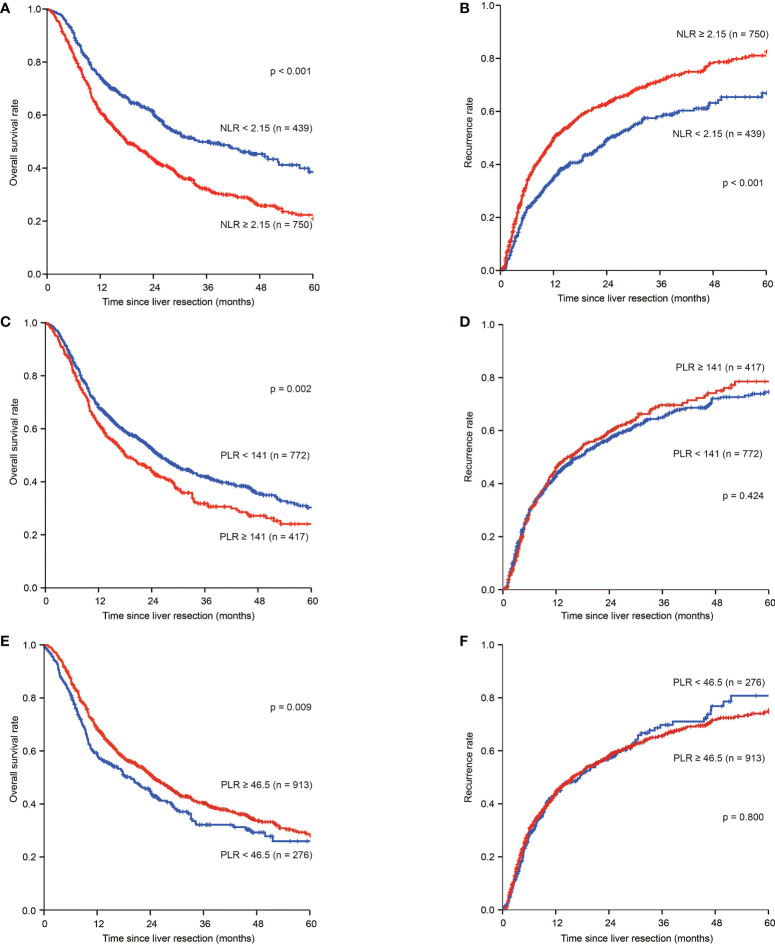
Kaplan–Meier estimate of OS and tumour recurrence for NLR ^high^ and NLR ^low^, PLR ^high^ and PLR ^low^, PNI ^high^ and PNI ^low^ group in the whole group. **(A)** Kaplan-Meier estimate of OS for ICC patients with NLR ^high^ and NLR ^low^ group; **(B)** Kaplan-Meier estimate of tumour recurrence for ICC patients with NLR ^high^ and NLR ^low^ group; **(C)** Kaplan-Meier estimate of OS for ICC patients with PLR ^high^ and PLR ^low^ group; **(D) **Kaplan-Meier estimate of tumour recurrence for ICC patients with PLR ^high^ and PLR ^low^ group; **(E)** Kaplan-Meier estimate of OS for ICC patients with PNI ^high^ and PNI ^low^ group; **(F)** Kaplan-Meier estimate of tumour recurrence for ICC patients with PNI ^high^ and PNI ^low^ group

For patients with NLR ^low^, the 1-, 3-, and 5-year OS rates were 74.2%, 48.7%, and 39.2%, respectively, which were significantly higher than those of patients with NLR ^high^ (61.1%, 33.5%, and 23.1%, respectively; *p*<0.001). The 1-, 3-, and 5-year tumour recurrence rates for patients in the NLR ^low^ group were 35.2%, 57.4%, and 65.4%, respectively, which were significantly lower than those observed in patients in the NLR ^high^ group (48.8%, 70.4%, and 80.5%, respectively; *p* < 0.001) (**Figures 1A, B**).

For patients in the PLR ^low^ group, the 1-, 3-, and 5-year OS rates were 68.1%, 42.5%, and 31.1%, respectively, and 61.9%, 32.6%, and 24.5%, respectively, for patients in the PLR ^high^ group (*p*=0.002). The 1-, 3-, and 5-year tumour recurrence rates for patients in the PLR ^low^ group were 43.2%, 64.6%, and 74.0%, respectively, while those for patients in the PLR ^high^ group were 44.8%, 67.9%, and 77.4%, respectively (*p*=0.424) (**Figures 1C, D**).

For patients in the PNI ^low^ group, the 1-, 3-, and 5-year OS rates were 58.3%, 32.9%, and 26.7%, respectively, and 68.2%, 40.9%, and 29.4%, respectively, for patients in the PNI ^high^ group (*p*=0.009) (**Figure 1E**). The 1-, 3-, and 5-year tumour recurrence rates for patients in the PNI ^low^ group were 42.5%, 68.2%, and 79.2%, respectively, while those for patients in the PNI ^high^ were 44.1%, 64.9%, and 74.1%, respectively (*p*=0.800) (**Figure 1F**).

The results of univariate analyses are presented in **Supplemental Table 1**. Multivariate analyses identified NLR ^high^ as an independent risk factor for OS and tumour recurrence (HR:1.327, 95% CI: 1.105–1.593; HR: 1.274, 95% CI: 1.074-1.510), in addition to CEA > 10 µg/L(HR: 1.432, 95% CI:1.154-1.776; HR: 1.309, 95% CI:1.049-1.635), CA 19-9 >39 U/L (HR: 1.396, 95% CI:1.186-1.644; HR: 1.237, 95% CI:1.052-1.455), tumour size ≥ 5 cm (HR: 1.300, 95% CI:1.093-1.547; HR: 1.389, 95% CI:1.170-1.649), multiple tumours (HR: 1.281, 95% CI: 1.084-1.514; HR: 1.470, 95% CI:1.244-1.737), adjacent organ invasion (HR: 1.731, 95% CI:1.316-2.277; HR: 1.551, 95% CI:1.144-2.104), lymph-node metastasis (HR: 1.463, 95% CI:1.210-1.768; HR: 1.246, 95% CI:1.023-1.518), and vascular invasion (HR: 1.420, 95% CI:1.188-1.699; HR: 1.471, 95% CI: 1.229-1.761) were independent risk factors of OS and tumour recurrence. Notably, PLR ^high^ or PNI ^high^ were not associated with OS and tumour recurrence compared to PLR ^low^ or PNI ^low^ (**Table 2**).

**Table 2:** Multivariate analysis of prognostic factors in ICC patients.

Variable	OS			Tumour recurrence		
	*P-value*	HR	95%CI	*P-value*	HR	95%CI
**Hepatolithiasis**, yes	0.431	1.090	0.880-1.349	–	–	–
**HBsAg**, yes	0.833	0.978	0.795-1.203	–	–	–
**HBcAb**, positive	0.518	0.934	0.759-1.149	–	–	–
**ALT**, U/L, >80	0.459	1.100	0.855-1.414	0.241	1.164	0.903-1.499
**CEA**, µg/L, >10	0.001	1.432	1.154-1.776	0.017	1.309	1.049-1.635
**CA 19-9**, U/L, >39	<0.001	1.396	1.186-1.644	0.010	1.237	1.052-1.455
**NLR**, ≥2.15	0.002	1.327	1.105-1.593	0.005	1.274	1.074-1.510
**PLR**, ≥141	0.743	1.029	0.866-1.224	–	–	–
**PNI**, ≥46.5	0.486	0.938	0.783-1.123	–	–	–
**Tumour size**, cm, ≥5	0.003	1.300	1.093-1.547	<0.001	1.389	1.170-1.649
**Tumour number**, multiple	0.004	1.281	1.084-1.514	<0.001	1.470	1.244-1.737
**Adjacent organs invasion**, yes	<0.001	1.731	1.316-2.277	0.005	1.551	1.144-2.104
**Lymph node metastasis**, yes	<0.001	1.463	1.210-1.768	0.029	1.246	1.023-1.518
**Vascular invasion**, yes	<0.001	1.420	1.188-1.699	<0.001	1.471	1.229-1.761

ICC, intrahepatic cholangiocarcinoma; OS, overall survival; HR, hazard ratio; CI, confidence interval; HBsAg, hepatitis B surface antigen; HBcAb, hepatitis B core antibody; ALT, alanine aminotransferase; CEA, carcinoembryonic antigen; CA 19-9, carbohydrate antigen 19-9; NLR, neutrophil to lymphocyte ratio; PLR, Platelet-Lymphocyte Ratio; PNI, prognostic nutritional index.

### Clinical characteristics of ICC patients with elevated NLR

Compared with patients with an NLR of < 2.15, those with an NLR of ≥ 2.15 were more likely to have higher levels of CA19-9 and CEA, larger tumour diameters, multiple tumours, lymph-node metastasis, vascular invasion, and more advanced tumours at TNM stage III/IV (**Table 1**).

### OS and tumour recurrence in patients with or without HBV infection

Among 788 and 401 patients who had or did not have HBV infection, the 1-, 3-, and 5-year OS rates were 67.6%, 41.3%, and 31.4%, respectively, for patients with HBV infection, and 62.6%, 34.5%, and 23.5%, respectively, for patients without HBV infection. The 1-, 3-, and 5-year tumour recurrence rates for patients with HBV infection were 44.6%, 65.4%, and 73.8%, respectively, whereas those for patients without HBV infection were 41.9%, 66.2%, and 78.2%, respectively.

Independent risk factors for tumour recurrence and OS were analysed in patients with or without HBV infection. The results of univariate analyses are shown in **Supplemental Tables 2**, **3**.

**Figure 2:**
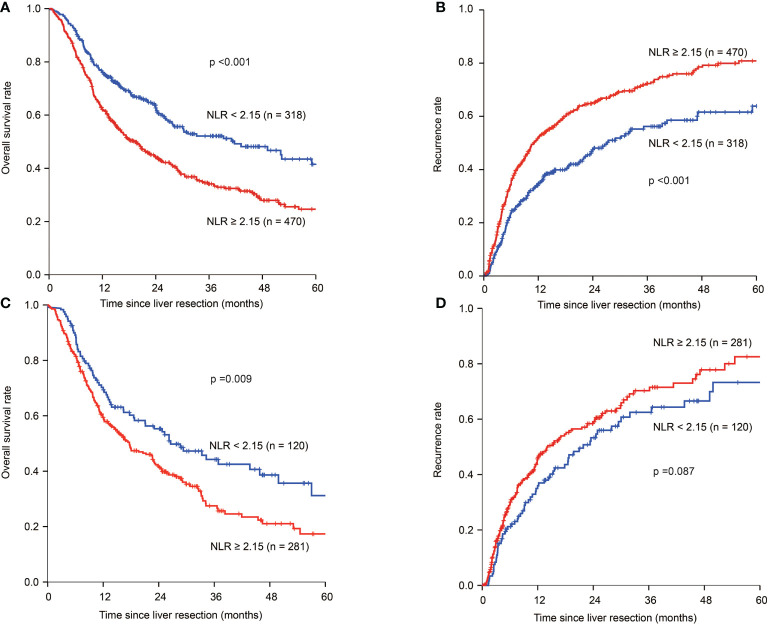
Kaplan–Meier estimate of OS and tumour recurrence for NLR ^high^ and NLR ^low^ ICC patients in the HBV infection group and no-HBV infection group. **(A)** Kaplan-Meier estimate of OS for NLR ^high^ and NLR ^low^ group in the HBV infection group. **(B)** Kaplan-Meier estimate of tumour recurrence for NLR ^high^ and NLR ^low^ group in the HBV infection group. **(C)** Kaplan-Meier estimate of OS for NLR ^high^ and NLR ^low^ group in the no-HBV infection group. **(D)** Kaplan-Meier estimate of tumour recurrence for NLR ^high^ and NLR ^low^ group in the no-HBV infection group.

In patients with HBV infection, the 1-, 3-, and 5-year OS rates were 76.0%, 52.2%, and 41.4%, respectively, for the NLR ^low^ patients, which were significantly higher than 62.0%, 34.5%, and 25.9%, respectively, for patients with NLR ^high^ (*p* < 0.001) (**Figure 2A**). The 1-, 3-, and 5-year tumour recurrence rates for patients in the NLR ^low^ group were 34.6%, 55.9%, and 62.7%, respectively, which were significantly lower than those for patients in the NLR ^high^ group (51.2%, 71.4%, and 80.2%, respectively; *p* < 0.001) (**Figure 2B**). Univariate analyses are shown in **Supplemental Table 2**. Multivariate analyses showed that NLR ≥2.15 (HR:1.347, 95% CI: 1.073-1.690; HR: 1.386, 95% CI: 1.112-1.726), tumour size ≥ 5 cm (HR:1.406, 95% CI: 1.129-1.750; HR: 1.484, 95% CI: 1.200–1.835), multiple tumours (HR:1.260, 95% CI: 1.024–1.552; HR: 1.416, 95% CI: 1.154–1.737), adjacent organ invasion (HR:1.627, 95% CI: 1.140–2.321; HR: 1.506, 95% CI: 1.034–2.192), lymph node metastasis (HR:1.424, 95% CI: 1.105–1.835; HR: 1.309, 95% CI: 1.012–1.694), and vascular invasion (HR: 1.388, 95% CI: 1.113–1.732; HR: 1.557, 95% CI: 1.256–1.931) were independent risk factors of OS and tumour recurrence. CA 19-9 >39 U/L (HR: 1.271, 95% CI: 1.040-1.551) was an independent risk factor for OS (**Table 3**).

**Table 3:** Multivariate analysis of prognostic factors in ICC patients with HBV infection.

Variable	OS			Tumour recurrence		
	*P*-value	HR	95%CI	*P*-value	HR	95%CI
**Hepatolithiasis**, yes	0.182	1.216	0.913-1.620	–	–	–
**AFP**, µg/L, >20	–	–	–	0.184	1.157	0.933-1.434
**CEA**, µg/L, >10	0.238	1.201	0.886-1.626	0.285	1.179	0.872-1.596
**CA 19-9**, U/L, >39	0.019	1.271	1.040-1.551	0.405	1.087	0.893-1.323
**NLR**, ≥2.15	0.010	1.347	1.073-1.690	0.004	1.386	1.112-1.726
**PLR**, ≥141	0.229	1.143	0.919-1.422	0.876	.983	0.793-1.219
**PNI**, ≥46.5	0.209	0.866	0.691-1.084	–	–	–
**Tumour size**, cm, ≥5	0.002	1.406	1.129-1.750	<0.001	1.484	1.200-1.835
**Tumour number**, multiple	0.029	1.260	1.024-1.552	0.001	1.416	1.154-1.737
**Adjacent organs invasion**, yes	0.007	1.627	1.140-2.321	0.033	1.506	1.034-2.192
**Lymph node metastasis**, yes	0.006	1.424	1.105-1.835	0.040	1.309	1.012-1.694
**Vascular invasion**, yes	0.004	1.388	1.113-1.732	<0.001	1.557	1.256-1.931

ICC, intrahepatic cholangiocarcinoma; HBV, hepatitis B virus; OS, overall survival; HR, hazard ratio; CI, confidence interval; AFP, a-fetoprotein; CEA, carcinoembryonic antigen; CA 19-9, carbohydrate antigen 19-9; NLR, neutrophil to lymphocyte ratio; PLR, Platelet-Lymphocyte Ratio; PNI, prognostic nutritional index.

In patients without HBV infection in the NLR ^low^ group, the 1-, 3-, and 5-year OS rates were 70.2%, 44.6%, and 32.4%, respectively, and 59.3%, 29.8%, and 19.0%, respectively, for those in the NLR ^high^ group (*p*=0.009) (**Figure 2C**). The 1-, 3-, and 5-year tumour recurrence rates for patients in the NLR ^low^ group were 35.9%, 61.5%, and 73.3%, respectively, while those for patients in the NLR ^high^ group were 44.6%, 68.6%, and 81.0%, respectively (*p*=0.087) (**Figure 2D**). Univariate analyses are shown in **Supplemental Table 3**. Multivariate analyses showed that CEA >10µg/L (HR: 1.673, 95% CI: 1.217-2.299; HR: 1.511, 95% CI: 1.075-2.125), CA 19-9 >39 U/L (HR: 1.665, 95% CI: 1.245-2.229; HR: 1.690, 95% CI: 1.257-2.272), multiple tumours (HR: 1.348, 95% CI: 1.019-1.782; HR: 1.580, 95% CI: 1.175-2.123), and adjacent organ invasion (HR: 2.054, 95% CI: 1.343-3.140; HR: 1.784, 95% CI: 1.059-3.005) were independent risk factors of OS and tumour recurrence. Lymph node metastasis (HR: 1.514, 95% CI: 1.139-2.011) and vascular invasion (HR: 1.679, 95% CI: 1.243-2.270) were independent risk factors for OS. NLR ≥ 2.15 was not an independent risk factor for OS and tumour recurrence in not HBV-infected ICC group (**Table 4**).

**Table 4:** Multivariate analysis of prognostic factors in ICC patients with no HBV infection.

Variable	OS			Tumour recurrence		
	*P*-value	HR	95%CI	*P*-value	HR	95%CI
**CEA**, µg/L, >10	0.002	1.673	1.217-2.299	0.018	1.511	1.075-2.125
**CA 19-9**, U/L, >39	0.001	1.665	1.245-2.229	0.001	1.690	1.257-2.272
**NLR**, ≥2.15	0.088	1.286	0.963-1.719	–	–	–
**Tumour size**, cm, ≥5	0.402	1.128	0.851-1.495	0.200	1.209	0.905-1.615
**Tumour number**, multiple	0.036	1.348	1.019-1.782	0.002	1.580	1.175-2.123
**Adjacent organs invasion**, yes	0.001	2.054	1.343-3.140	0.030	1.784	1.059-3.005
**Lymph node metastasis**, yes	0.004	1.514	1.139-2.011	0.249	1.203	0.878-1.648
**Vascular invasion**, yes	0.001	1.679	1.243-2.270	0.087	1.336	0.959-1.861

ICC, intrahepatic cholangiocarcinoma; HBV, hepatitis B virus; OS, overall survival; HR, hazard ratio; CI, confidence interval; CEA, carcinoembryonic antigen; CA 19-9, carbohydrate antigen 19-9; NLR, neutrophil to lymphocyte ratio.

### Basic clinical features for HBV-ICC patients with elevated NLR

Subgroup analysis was performed for patients with HBV-ICC. Compared with patients with a NLR of < 2.15, patients with a NLR of ≥ 2.15 were more likely to have higher levels of CA19-9, larger tumour diameters, multiple tumours, lymph-node metastasis, more advanced tumours at TNM stage III/IV, and more non-cirrhosis (**Table 5**).

**Table 5:** Baseline clinicopathological data for HBV-ICC Patients.

Variable	Number (%)/median (IQR)			*P*-value
	Total (n = 788)	NLR < 2.15 (n = 318)	NLR ≥ 2.15 (n = 470)
**Age**, years	54.0 (46.0-60.0)	54.0 (46.0-59.0)	54.0 (46.0-61.0)	0.905
**Sex**,				
**Female**	275 (34.9%)	107 (33.6%)	168 (35.7%)	0.545
**Male**	513 (65.1%)	211 (66.4%)	302 (64.3%)	
**Hepatolithiasis**,				
**NO**	697 (88.5%)	277(87.1%)	420 (89.4%)	0.331
**Yes**	91 (11.5%)	41 (12.9%)	50 (10.6%)	
**Anti-HCV**				
**Negative**	776 (98.5%)	313 (98.4%)	463(98.5%)	0.926
**Positive**	12 (1.5%)	5 (1.6%)	7 (1.5%)	
**TBIL**, µmol/L	12.6 (9.7-16.7)	12.4 (9.8-16.2)	12.8 (9.6-17.2)	0.272
**ALB**, g/L	42.4 (39.9-44.9)	42.6 (40.5-44.7)	42.4 (39.5-44.9)	0.258
**ALT**, U/L	28.0 (18.6-43.6)	30.0 (20.0-42.6)	26.6 (17.5-44.9)	0.112
**PT,** seconds	11.7 (11.2-12.4)	11.7 (11.2-12.4)	11.7 (11.2-12.3)	0.664
**AFP**, µg/L	4.0 (2.4-16.6)	4.0 (2.4-11.5)	4.0 (2.3-20.0)	0.538
**CEA**, µg/L	2.5 (1.6-4.3)	2.3 (1.6-3.8)	2.7 (1.6-4.6)	0.058
**CA 19-9**, U/mL	39.2 (15.8-135.6)	30.9 (14.0-75.1)	49.6 (16.8-220.3)	<0.001
**PLR**	111.1 (82.4-146.4)	87.6 (69.4-111.9)	130.7 (98.7-167.5)	<0.001
**PNI**	50.4 (46.9-53.7)	52.2 (48.5-55.3)	49.5 (46.1-52.4)	<0.001
**Operation time**, hours	2.0 (1.5-2.5)	2.0 (1.5-2.5)	2.0 (1.5-2.5)	0.071
**Hilar clamping**, minutes	15.0 (9.0-22.0)	15.0 (9.0-21.0)	16.0 (8.0-22.0)	0.177
**Gross type**				
**Mass-forming**	774 (98.2%)	311 (97.8%)	463 (98.5%)	0.458
**No mass-forming**	14 (1.8%)	7 (2.2%)	7 (1.5%)	
**Cirrhosis**				
**No**	560 (71.1%)	195 (61.3%)	365 (77.7%)	<0.001
**Yes**	228 (28.9%)	123 (38.7%)	105 (22.3%)	
**Tumour diameter**, cm	5.6 (4.0-8.0)	4.5 (3.5-6.2)	6.4 (4.6-9.0)	<0.001
**Tumour number**				
**Solitary**	560 (71.1%)	245 (77.0%)	315 (67.0%)	0.002
**Multiple**	228 (28.9%)	73 (23.0%)	155 (33.0%)	
**Adjacent organs invasion**				
**No**	738 (93.7%)	301 (94.7%)	437 (93.0%)	0.344
**Yes**	50 (6.3%)	17 (5.3%)	33 (7.0%)	
**Lymph node metastasis**				
**No**	672 (85.3%)	285 (89.6%)	387 (82.3%)	0.005
**Yes**	116 (14.7%)	33 (10.4%)	83 (17.7%)	
**Vascular invasion**				
**No**	623 (79.1%)	260 (81.8%)	363 (77.2%)	0.125
**Yes**	165 (20.9%)	58 (18.2%)	107 (22.8%)	
**Differentiation**				
**Poor**	42 (5.3%)	21 (6.6%)	21 (4.5%)	0.190
**Moderate/Well**	746 (94.7%)	297 (93.4%)	449 (95.5%)	
**TNM**				
**I/II**	639 (81.1%)	272 (85.5%)	367 (78.1%)	0.009
**III/IV**	149 (18.9%)	46 (14.5%)	103 (21.9%)	

HBV, hepatitis B virus; ICC, intrahepatic cholangiocarcinoma; IQR, interquartile range; HBsAg, hepatitis B surface antigen; HBeAg, hepatitis Be Antigen; HBcAb, hepatitis B core antibody; HCV, hepatitis C virus; TBIL, total bilirubin; ALB, Albumin; ALT, alanine aminotransferase; PT, prothrombin time; AFP, a-fetoprotein; CEA, carcinoembryonic antigen; CA 19-9, carbohydrate antigen 19-9; NLR, neutrophil to lymphocyte ratio; PLR, Platelet-Lymphocyte Ratio; PNI, prognostic nutritional index; TNM, tumour node metastasis.

### OS and tumour recurrence in HBV-ICC patient in the propensity score matching cohort

After PSM, 430 patients from the two groups were selected for further subset analysis. The baseline demographic and laboratory features were not significantly different between patients with NLR < 2.15 and ≥ 2.15 (**Table 6**).

**Table 6:** Baseline clinicopathological data for HBV-ICC Patients in the PSM cohort.

Variable	Number (%)/median (IQR)			*P*-value
	Total (n = 430)	NLR < 2.15 (n = 215)	NLR ≥ 2.15 (n = 215)
**Age**, years	54.0 (46.0-60.3)	55.0 (47.0-60.0)	54.0 (46.0-61.0)	0.337
**Sex**,				
**Female**	157 (36.5%)	75 (34.9%)	82 (38.1%)	0.483
**Male**	273 (63.5%)	140 (65.1%)	133 (61.9%)	
**Hepatolithiasis**,				
**NO**	376 (87.4%)	183(85.1%)	193 (89.8%)	0.146
**Yes**	54 (12.6%)	32 (14.9%)	22 (10.2%)	
**Anti-HCV**				
**Negative**	426 (99.1%)	213(99.1%)	213 (99.1%)	1.000
**Positive**	4 (0.9%)	2 (0.9%)	2 (0.9%)	
**TBIL**, µmol/L	12.5 (9.8-16.0)	12.2 (9.6-14.9)	13.0(10.0-17.0)	0.069
**ALB**, g/L	42.6 (40.6-45.1)	42.0 (40.0-44.4)	42.2(40.9-43.6)	0.131
**ALT**, U/L	27.9 (19.0-41.5)	27.3 (19.0-38.8)	28.0 (18.9-44.5)	0.721
**PT,** seconds	11.7 (11.2-12.3)	11.6 (11.2-12.4)	11.7 (11.2-12.2)	0.752
**AFP**, µg/L	3.8 (2.4-12.0)	3.7 (2.3-8.4)	4.0 (2.4-21.4)	0.107
**CEA**, µg/L	2.4 (1.6-4.2)	2.3 (1.5-3.9)	2.6 (1.7-4.5)	0.197
**CA 19-9**, U/mL	36.3 (15.7-114.2)	33.9 (14.9-90.2)	40.6 (6.3-139.8)	0.186
**PLR**	102.1 (80.7-127.8)	98.8 (80.3-123.7)	105.9 (81.0-134.7)	0.063
**PNI**	51.4 (47.8-54.0)	51.7 (48.1-54.1)	51.3 (47.8-54.0)	0.673
**Operation time**, hours	2.0 (1.5-2.5)	2.0 (1.5-2.5)	1.9(1.5-2.5)	0.412
**Hilar clamping**, minutes	15.0 (8.0-22.0)	15.0 (7.0-21.0)	16.0 (9.0-23.0)	0.188
**Gross type**				
**Mass-forming**	423 (98.4%)	210 (97.7%)	213 (99.1%)	0.449
**No mass-forming**	7 (1.6%)	5 (2.3%)	2 (0.9%)	
**Cirrhosis**				
**No**	302 (70.2%)	153 (71.2%)	149 (69.3%)	0.673
**Yes**	128 (29.8%)	62 (28.8%)	66 (30.7%)	
**Tumour diameter**, cm	5.1 (3.9-7.2)	5.0 (3.8-7.0)	5.2 (4.0-7.4)	0.195
**Tumour number**				
**Solitary**	332 (77.2%)	165 (76.7%)	167 (77.7%)	0.818
**Multiple**	98 (22.8%)	50 (23.3%)	48 (22.3%)	
**Adjacent organs invasion**				
**No**	405 (94.2%)	203 (94.4%)	202 (94.0%)	0.837
**Yes**	25 (5.8%)	12 (5.6%)	13 (6.0%)	
**Lymph node metastasis**				
**No**	374 (87.0%)	190 (88.4%)	184 (85.6%)	0.390
**Yes**	56 (13.0%)	25 (11.6%)	31 (14.4%)	
**Vascular invasion**				
**No**	343 (79.8%)	176 (81.9%)	167 (77.7%)	0.280
**Yes**	87 (20.2%)	39 (18.1%)	48 (22.3%)	
**Differentiation**				
**Poor**	18 (4.2%)	13 (6.0%)	5 (2.3%)	0.054
**Moderate/Well**	412(95.8%)	202 (94.0%)	210 (97.7%)	
**TNM**				
**I/II**	355 (82.6%)	180 (83.7%)	175 (81.4%)	0.525
**III/IV**	75 (17.4%)	35 (16.3%)	40 (18.6%)	

HBV, hepatitis B virus; ICC, intrahepatic cholangiocarcinoma; PSM, propensity score matching; IQR, interquartile range; HBsAg, hepatitis B surface antigen; HBeAg, hepatitis Be Antigen; HBcAb, hepatitis B core antibody; HCV, hepatitis C virus; TBIL, total bilirubin; ALB, Albumin; ALT, alanine aminotransferase; PT, prothrombin time; AFP, a-fetoprotein; CEA, carcinoembryonic antigen; CA 19-9, carbohydrate antigen 19-9; NLR, neutrophil to lymphocyte ratio; PLR, Platelet-Lymphocyte Ratio; PNI, prognostic nutritional index; TNM, tumour node metastasis.

The independent risk factors for OS and tumour recurrence were analysed in patients with NLR < 2.15 vs. ≥ 2.15 after PSM. Univariate analyses are shown in **Supplemental Table 4**. Multivariate analyses showed that NLR ≥2.15 (HR: 1.421, 95% CI: 1.074–1.881; HR: 1.358, 95% CI: 1.029–1.792), CEA >10µg/L (HR: 1.915, 95% CI: 1.230–2.979; HR: 1.829, 95% CI: 1.178–2.842), tumour size ≥ 5 cm (HR: 1.455, 95% CI: 1.088–1.947; HR: 1.488, 95% CI: 1.111–1.992), multiple tumours (HR: 1.567, 95% CI: 1.146–2.142; HR: 1.479, 95% CI: 1.085–2.018), adjacent organ invasion (HR: 1.770, 95% CI: 1.062–2.951; HR: 1.894, 95% CI: 1.138–3.149), and vascular invasion (HR: 1.861, 95% CI: 1.348–2.568; HR: 1.855, 95% CI: 1.346–2.556) were independent risk factors of OS and tumour recurrence. Moderate/well differentiation (HR: 0.419, 95% CI: 0.231–0.762) was an independent protective factor for OS (**Table 7**).

**Table 7:** Multivariate analysis of prognostic factors in ICC patients with HBV infection in the PSM cohort.

Variable	OS			Tumour recurrence		
	*P*-value	HR	95%CI	*P*-value	HR	95%CI
**PT**, seconds, >13	0.325	1.289	0.778-2.136	0.232	1.361	0.821-2.256
**AFP**, µg/L, >20	–	–	–	0.711	1.064	0.766-1.479
**CEA**, µg/L, >10	0.004	1.915	1.230-2.979	0.007	1.829	1.178-2.842
**CA 19-9**, U/L, >39	0.081	1.285	0.969-1.703	0.122	1.247	0.942-1.651
**NLR**, ≥2.15	0.014	1.421	1.074-1.881	0.030	1.358	1.029-1.792
**Tumour size**, cm, ≥5	0.011	1.455	1.088-1.947	0.008	1.488	1.111-1.992
**Tumour number**, multiple	0.005	1.567	1.146-2.142	0.013	1.479	1.085-2.018
**Adjacent organs invasion**, yes	0.028	1.770	1.062-2.951	0.014	1.894	1.138-3.149
**Lymph node metastasis**, yes	0.429	1.167	0.796-1.713	0.522	1.134	0.772-1.667
**Differentiation**, moderate / well	0.004	0.419	0.231-0.762	–	–	–
**Vascular invasion**, yes	<0.001	1.861	1.348-2.568	<0.001	1.855	1.346-2.556

ICC, intrahepatic cholangiocarcinoma; HBV, hepatitis B virus; OS, overall survival; HR, hazard ratio; CI, confidence interval; PT, prothrombin time; AFP, a-fetoprotein; CEA, carcinoembryonic antigen; CA 19-9, carbohydrate antigen 19-9; NLR, neutrophil to lymphocyte ratio; PLR, Platelet-Lymphocyte Ratio; PNI, prognostic nutritional index.

## Discussion

Increasing evidence has shown that inflammatory cells and factors play important roles in tumour progression. In the eighteenth century, Virchow first observed leukocytes in neoplastic tissues (25), which led to the hypothesis that inflammation plays an important role in the development of malignant disease. This hypothesis has been extensively tested. Studies have shown that the inflammation marker NLR was associated with the prognosis of many solid tumours (10–18), especially in colorectal cancer, indicating that inflammation markers have important prognostic value (11–13). However, their role in liver cancer is not completely understood. In ICC, there have been few studies regarding NLR.

To the best of our knowledge, the role of inflammatory markers in ICC patients with different causative factors has not yet been reported. A controlled case study in China suggested that HBV infection was a risk factor for ICC (26) and could lead to inflammatory responses. ICC caused by HBV infection differs in many aspects. For example, patients with HBV infections had higher lymphocyte counts and ratios and lower neutrophil counts and ratios than uninfected patients. Lymphocytes reflect autoimmune inflammation, whereas neutrophils reflect acute inflammation. In addition, patients with HBV infections exhibited lower NLR, while NLR value was much higher in patients without HBV infection. To explore the roles of NLR, PLR, and PNI in ICC, we analysed the correlation of preoperative inflammation markers with OS and tumour recurrence in ICC patients who underwent hepatic resection. We found that only NLR was associated with OS and tumour recurrence. Elevated NLR indicated early recurrence, high tumour recurrence, and shorter survival, suggesting that it has more prognostic value than PNI and PLR, which is consistent with the reports of Proctor et al. (27) and Dirican et al. (28). Moreover, subgroup analysis showed that elevated NLR was an independent risk factor for OS and tumour recurrence in patients with HBV infection. In addition, we performed PSM matching in patients with HBV-ICC; this analysis showed that NLR remained an independent risk factor for OS and TTR. In the non-HBV-infection group, inflammatory markers were not independent risk factors for OS and tumour recurrence.

Further analysis of the possible reasons for the poor prognosis of patients with with NLR ≥ 2.15 showed that their neutrophil counts were significantly higher than in patients with NLR < 2.15 (5.19×10^9^/L vs. 3.37×10^9^/L), while lymphocyte counts were significantly lower in patients with NLR ≥ 2.15 than in patients with NLR < 2.15 (1.47×10^9^/L vs. 2.02×10^9^/L), consistent with a previous report (29). Increased neutrophil counts represent acute inflammation; the higher the neutrophil count, the more serious the inflammation. In contrast, increased lymphocyte counts represent the inflammatory response of the immune system; the higher of the lymphocyte count, the stronger the immune system. This result suggests that the acute inflammatory response may drive tumour metastasis, whereas lymphocytes from the innate immune system may be protective. Patients with NLR ≥ 2.15 had higher CA19-9 and CEA levels, larger tumour diameters, more tumour nodes, and more severe node metastasis and vascular invasion. Moreover, more patients with NLR ≥ 2.15 presented with TNM stage III/IV. The results showed that tumour invasion was greater in patients with NLR ≥ 2.15.

The specific reasons for decreased OS and higher tumour recurrence in patients with elevated NLR are not well understood. Our study suggests that these phenomena may be associated with acute inflammation, innate immunity, and tumour biology. Previous studies have shown that elevated NLR is associated with systemic inflammation, decreased lymphocyte count, and damaged innate anti-tumour immunity (30–33). In addition, increased neutrophil levels are regarded as reservoirs of vascular endothelial growth factor (VEGF) (34), which plays an important role in tumour development and metastasis. Experimental studies have shown direct and indirect interactions between host inflammatory cells and the primary tumour, and the subsequent promotion of angiogenesis, extracellular matrix remodelling, and the metastatic niche (35, 36). In addition, the numbers of T cells in tumours are associated with their growth and metastasis (37). Moreover, lymphocytes can resist tumour cells and antagonise the role of monocytes in tumour development (38). Some researchers hypothesise that tumours can lead to inflammatory responses by increasing cytokines, inflammatory mediators, and inflammatory bodies, which further promotes tumour recurrence and metastasis by inhibiting apoptosis, promoting angiogenesis, and destroying DNA (39–41).

The study of serum inflammatory markers in hepatobiliary tumors is hot. Previous literatures reported the importance of serum inflammatory markers in the prognosis of hepatocellular carcinoma (HCC) and ICC patients (20, 42). Compared with a previous study on inflammatory markers of ICC (20), our study had a larger sample size, which allowed us to more specifically study the biology of ICC and the association of inflammatory markers with prognosis. To the best of our knowledge, our study is the first study to investigate the relationship between inflammation markers and prognosis in ICC patients according to HBV infection or not. Our study revealed the significant prognostic value of inflammatory markers and could help in selecting postoperative adjuvant treatment for patients with ICC and HBV infection. This study is a retrospective analysis of the prognostic value of inflammatory markers in ICC patients with different causes. Thus, these results need to be validated further.

## Conclusions

We found that elevated NLR significantly increased the risk of tumour recurrence and death in ICC patients who underwent liver resection, especially for HBV-ICC. In addition, the NLR is correlated with tumour invasion. Patients with NLR ≥ 2.15 showed higher CA19-9 and CEA levels, larger tumour diameters, multiple tumours, as well as more severe lymph node metastasis and vascular invasion. More patients with elevated NLR presented with TNM stage III/IV. Since NLR can be obtained during routine examinations before surgery, it can be used to predict prognosis and help select postoperative adjuvant treatments. With advancing studies regarding inflammatory responses in ICC patients, NLR may become a potential target for ICC treatment.

## Data availability statement

The data analyzed in this study is subject to the following licenses/restrictions: (The data are not publicly available due to privacy or ethical restrictions). Requests to access these datasets should be directed to [Kui Wang/Department of Hepatic Surgery (II), Eastern Hepatobiliary Surgery Hospital, Navy Medical University, Shanghai, China. + E-mail: wangkuiykl@163.com].

## Ethics statement

Written informed consent was obtained from the individual(s) for the publication of any potentially identifiable images or data included in this article.

## Author contributions

JL, YX, and FX contributed to the study concept, data collection, analysis, and drafting of the manuscript. CL, JW, CW, YW, and SB contributed to data collection, follow-up of patients, and analysis. FS and KW contributed to the study concept, design, drafting of the manuscript, and study supervision. All authors contributed to the article and approved the submitted version.

## Funding

This study was funded in full by the Program of Science and Technology Commission of Shanghai Municipality (grant number 21Y11912700), Natural Science and Medical Guidance Foundation of Shanghai (grant number 16ZR1400100 and 16411966200), the National Natural Science Foundation of China, Youth Science Fund Project (grant number 31301187), Clinical Research Plan for SHDC (grant number SHDC2020CR2038B), Explorer Program of Shanghai Scientific and Technological Committee (grant number 21TS1400500), Clinical specialist project in Shanghai (grant number shslczdzk02402), Project of Shanghai Shenkang Hospital Development Center (grant number SHDC2020CR5007, SHDC12019110)

## Conflict of interest

The authors declare that the research was conducted in the absence of any commercial or financial relationships that could be construed as a potential conflict of interest.

## Publisher’s note

All claims expressed in this article are solely those of the authors and do not necessarily represent those of their affiliated organizations, or those of the publisher, the editors and the reviewers. Any product that may be evaluated in this article, or claim that may be made by its manufacturer, is not guaranteed or endorsed by the publisher.

## Supplementary material

Supplementary Figure 1 Calculation process of the minimum *p*-value of the cut-off values of neutrophil-lymphocyte ratio, platelet-lymphocyte ratio, and prognostic nutritional index.
