# Supplementary material for: Elevated serum neutrophil-lymphocyte ratio is associated with worse long-term survival in patients with HBV-related intrahepatic cholangiocarcinoma undergoing resection
Source: Front Oncol. 2022 Oct 17;12:1012246. doi: 10.3389/fonc.2022.1012246 (PMC9618718; doi:10.3389/fonc.2022.1012246)
Supplement: Supplementary file 1 [file Table_1.docx]

| **Supplemental Table 1. Univarate analysis of prognostic factors in ICC patients** | | | | | | |
| --- | --- | --- | --- | --- | --- | --- |
| **Variable** | **OS** | | | **Tumour recurrence** | | |
|  | ***P-value*** | **HR** | **95%CI** | ***P-value*** | **HR** | **95%CI** |
| **Age**, years, >60 | 0.055 | 1.170 | 0.996-1.373 | 0.748 | 0.973 | 0.826-1.148 |
| **Sex**, male | 0.378 | 1.074 | 0.916-1.261 | 0.715 | 1.030 | 0.879-1.208 |
| **Hepatolithiasis**, yes | 0.045 | 1.237 | 1.005-1.522 | 0.825 | 0.976 | 0.784-1.214 |
| **HBsAg**, positive | 0.012 | 0.824 | 0.709-0.958 | 0.912 | 1.009 | 0.868-1.172 |
| **HBeAg**, positive | 0.702 | 1.047 | 0.826-1.328 | 0.325 | 1.125 | 0.890-1.423 |
| **HBcAb**, positive | 0.047 | 0.854 | 0.731-0.998 | 0.672 | 1.035 | 0.882-1.215 |
| **Anti-HCV**, positive | 0.194 | 1.404 | 0.841-2.343 | 0.877 | 1.046 | 0.591-1.852 |
| **TBIL**, µmol/L, >17 | 0.698 | 0.965 | 0.807-1.154 | 0.688 | 0.964 | 0.807-1.152 |
| **ALB**, g/L, ≥35 | 0.274 | 0.840 | 0.615-1.148 | 0.368 | 1.174 | 0.828-1.667 |
| **ALT**, U/L, >80 | 0.044 | 1.283 | 1.006-1.636 | 0.023 | 1.333 | 1.040-1.708 |
| **PT**, seconds, >13 | 0.267 | 1.167 | 0.888-1.535 | 0.094 | 1.261 | 0.961-1.654 |
| **AFP**, µg/L, >20 | 0.711 | 0.963 | 0.790-1.175 | 0.062 | 1.199 | 0.991-1.451 |
| **CEA**, µg/L, >10 | <0.001 | 2.245 | 1.849-2.725 | <0.001 | 1.828 | 1.484-2.250 |
| **CA 19-9**, U/L, >39 | <0.001 | 1.815 | 1.556-2.118 | <0.001 | 1.561 | 1.340-1.819 |
| **NLR** ≥2.15 | <0.001 | 1.651 | 1.400-1.948 | <0.001 | 1.549 | 1.317-1.823 |
| **PLR** ≥141 | 0.002 | 1.280 | 1.097-1.494 | 0.424 | 1.066 | 0.911-1.249 |
| **PNI** ≥46.5 | 0.009 | 0.796 | 0.671-0.946 | 0.800 | 0.977 | 0.817-1.169 |
| **Operation time**, hours, ≥3 | 0.078 | 1.173 | 0.982-1.400 | 0.630 | 0.956 | 0.795-1.149 |
| **Hilar clamping**, minutes, ≥30 | 0.546 | 0.921 | 0.705-1.203 | 0.488 | 1.093 | 0.850-1.404 |
| **Gross type**, no mass-forming | 0.785 | 1.074 | 0.643-1.792 | 0.072 | 0.527 | 0.262-1.058 |
| **Cirrhosis**, yes | 0.994 | 0.999 | 0.831-1.202 | 0.788 | 1.026 | 0.853-1.234 |
| **Tumour size** ≥ 5 cm | <0.001 | 1.678 | 1.424-1.976 | <0.001 | 1.694 | 1.440-1.993 |
| **Tumour number**, multiple | <0.001 | 1.637 | 1.396-1.919 | <0.001 | 1.797 | 1.531-2.109 |
| **Adjacent organs invasion**, yes | <0.001 | 2.799 | 2.1743.603 | <0.001 | 2.265 | 1.694-3.028 |
| **Lymph node metastasis**, yes | <0.001 | 1.958 | 1.636-2.343 | <0.001 | 1.604 | 1.326-1.940 |
| **Vascular invasion**, yes | <0.001 | 1.660 | 1.396-1.974 | <0.001 | 1.686 | 1.412-2.012 |
| **Differentiation**, moderate/well | 0.364 | 0.854 | 0.608-1.200 | 0.851 | 0.966 | 0.674-1.385 |
| **TNM**, III/IV | <0.001 | 1.690 | 1.427-2.002 | <0.001 | 1.423 | 1.193-1.698 |
| **Abbreviation:** ICC, intrahepatic cholangiocarcinoma; OS, overall survival; HR, hazard ratio; CI, confidence interval; HBsAg, hepatitis B surface antigen; HBeAg, hepatitis Be Antigen; HBcAb, hepatitis B core antibody; HCV, hepatitis C virus; TBIL, total bilirubin; ALB, Albumin; ALT, alanine aminotransferase; PT, prothrombin time; AFP, a-fetoprotein; CEA, carcinoembryonic antigen; CA 19-9, carbohydrate antigen 19-9; NLR, neutrophil to lymphocyte ratio; PLR, Platelet-Lymphocyte Ratio; PNI, prognostic nutritional index; TNM, tumour node metastasis. | | | | | | |
