# Supplementary material for: Elevated serum neutrophil-lymphocyte ratio is associated with worse long-term survival in patients with HBV-related intrahepatic cholangiocarcinoma undergoing resection
Source: Front Oncol. 2022 Oct 17;12:1012246. doi: 10.3389/fonc.2022.1012246 (PMC9618718; doi:10.3389/fonc.2022.1012246)
Supplement: Supplementary file 2 [file Table_2.docx]

| **Supplemental Table 2. Univariate analysis of prognostic factors in ICC patients with HBV infection** | | | | | | |
| --- | --- | --- | --- | --- | --- | --- |
| **Variable** | **OS** | | | **Tumour recurrence** | | |
|  | ***P*-value** | **HR** | **95%CI** | ***P*-value** | **HR** | **95%CI** |
| **Age**, years, >60 | 0.310 | 1.118 | 0.902-1.385 | 0.247 | 0.880 | 0.709-1.092 |
| **Sex**, male | 0.779 | 1.029 | 0.845-1.253 | 0.826 | 0.979 | 0.809-1.185 |
| **Hepatolithiasis**, yes | 0.038 | 1.344 | 1.017-1.777 | 0.946 | 1.010 | 0.754-1.352 |
| **Anti-HCV**, positive | 0.334 | 1.412 | 0.701-2.844 | 0.757 | 0.881 | 0.393-1.972 |
| **TBIL**, µmol/L, >17 | 0.791 | 1.030 | 0.828-1.280 | 0.916 | 1.012 | 0.817-1.252 |
| **ALB**, g/L, ≥35 | 0.273 | 0.804 | 0.544-1.188 | 0.916 | 0.978 | 0.654-1.465 |
| **ALT**, U/L, >80 | 0.113 | 1.285 | 0.942-1.751 | 0.156 | 1.260 | 0.915-1.733 |
| **PT**, seconds, >13 | 0.155 | 1.246 | 0.920-1.687 | 0.106 | 1.280 | 0.949-1.727 |
| **AFP**, µg/L, >20 | 0.701 | 1.044 | 0.838-1.300 | 0.029 | 1.262 | 1.024-1.555 |
| **CEA**, µg/L, >10 | <0.001 | 2.017 | 1.539-2.644 | <0.001 | 1.711 | 1.293-2.266 |
| **CA 19-9**, U/L, >39 | <0.001 | 1.620 | 1.340-1.957 | <0.001 | 1.398 | 1.163-1.681 |
| **NLR**, ≥2.15 | <0.001 | 1.733 | 1.413-2.125 | <0.001 | 1.696 | 1.393-2.065 |
| **PLR**, ≥141 | <0.001 | 1.472 | 1.206-1.797 | 0.020 | 1.266 | 1.038-1.544 |
| **PNI**, ≥46.5 | 0.003 | 0.722 | 0.582-0.895 | 0.071 | 0.819 | 0.659-1.017 |
| **Operation time**, hours, ≥3 | 0.259 | 1.153 | 0.900-1.477 | 0.776 | 1.036 | 0.811-1.324 |
| **Hilar clamping**, minutes, ≥30 | 0.949 | 1.011 | 0.722-1.416 | 0.218 | 1.216 | 0.891-1.660 |
| **Gross type**, no mass-forming | 0.626 | 1.179 | 0.608-2.283 | 0.528 | 0.771 | 0.344-1.728 |
| **Cirrhosis**, yes | 0.779 | 1.030 | 0.838-1.266 | 0.761 | 1.032 | 0.843-1.262 |
| **Tumour size**, cm, ≥5 | <0.001 | 1.828 | 1.488-2.246 | <0.001 | 1.861 | 1.526-2.271 |
| **Tumour number**, multiple | <0.001 | 1.630 | 1.338-1.987 | <0.001 | 1.754 | 1.444-2.130 |
| **Adjacent organs invasion**, yes | <0.001 | 2.544 | 1.834-3.527 | <0.001 | 2.140 | 1.499-3.056 |
| **Lymph node metastasis**, yes | <0.001 | 1.884 | 1.482-2.396 | <0.001 | 1.677 | 1.313-2.142 |
| **Vascular invasion**, yes | <0.001 | 1.884 | 1.482-2.396 | <0.001 | 1.821 | 1.475-2.248 |
| **Differentiation**, moderate/well | 0.101 | 0.729 | 0.500-1.063 | 0.428 | 0.852 | 0.573-1.266 |
| **TNM**, III/IV | <0.001 | 1.748 | 1.404-2.177 | <0.001 | 1.573 | 1.260-1.963 |
| **Abbreviation**: ICC, intrahepatic cholangiocarcinoma; HBV, hepatitis B virus; OS, overall survival; HR, hazard ratio; CI, confidence interval; HCV, hepatitis C virus; TBIL, total bilirubin; ALB, Albumin; ALT, alanine aminotransferase; PT, prothrombin time; AFP, a-fetoprotein; CEA, carcinoembryonic antigen; CA 19-9, carbohydrate antigen 19-9; NLR, neutrophil to lymphocyte ratio; PLR, Platelet-Lymphocyte Ratio; PNI, prognostic nutritional index; TNM, tumour node metastasis. | | | | | | |
