# Supplementary material for: Elevated serum neutrophil-lymphocyte ratio is associated with worse long-term survival in patients with HBV-related intrahepatic cholangiocarcinoma undergoing resection
Source: Front Oncol. 2022 Oct 17;12:1012246. doi: 10.3389/fonc.2022.1012246 (PMC9618718; doi:10.3389/fonc.2022.1012246)
Supplement: Supplementary file 3 [file Table_3.docx]

| **Supplemental Table 3. Univariate analysis of prognostic factors in ICC patients with no HBV infection** | | | | | | |
| --- | --- | --- | --- | --- | --- | --- |
| **Variable** | **OS** | | | **Tumour recurrence** | | |
|  | ***P*-value** | **HR** | **95%CI** | ***P*-value** | **HR** | **95%CI** |
| **Age**, years, >60 | 0.229 | 1.167 | 0.907-1.502 | 0.307 | 1.150 | 0.880-1.502 |
| **Sex**, male | 0.257 | 1.173 | 0.890-1.544 | 0.280 | 1.174 | 0.878-1.571 |
| **Hepatolithiasis**, yes | 0.747 | 1.053 | 0.769-1.442 | 0.715 | 0.940 | 0.673-1.313 |
| **Anti-HCV**, positive | 0.477 | 1.314 | 0.619-2.788 | 0.508 | 1.316 | 0.584-2.963 |
| **TBIL**, µmol/L, >17 | 0.371 | 0.866 | 0.631-1.187 | 0.357 | 0.858 | 0.619-1.189 |
| **ALB**, g/L, ≥35 | 0.708 | 0.905 | 0.537-1.526 | 0.112 | 1.774 | 0.875-3.597 |
| **ALT**, U/L, >80 | 0.306 | 1.229 | 0.829-1.821 | 0.068 | 1.449 | 0.973-2.156 |
| **PT**, seconds, >13 | 0.818 | 1.081 | 0.555-2.104 | 0.655 | 1.165 | 0.597-2.272 |
| **AFP**, µg/L, >20 | 0.444 | 0.789 | 0.431-1.446 | 0.566 | 0.830 | 0.439-1.569 |
| **CEA**, µg/L, >10 | <0.001 | 2.446 | 1.837-3.257 | <0.001 | 2.105 | 1.530-2.896 |
| **CA 19-9**, U/L, >39 | <0.001 | 2.241 | 1.707-2.941 | <0.001 | 2.033 | 1.537-2.690 |
| **NLR**, ≥2.15 | 0.009 | 1.456 | 1097-1.933 | 0.087 | 1.289 | 0.964-1.723 |
| **PLR**, ≥141 | 0.806 | 0.969 | 0.754-1.245 | 0.132 | 0.815 | 0.625-1.064 |
| **PNI**, ≥46.5 | 0.800 | 0.964 | 0.725-1.282 | 0.054 | 1.368 | 0.995-1.880 |
| **Operation time**, hours, ≥3 | 0.432 | 1.112 | 0.854-1.448 | 0.331 | 0.868 | 0.652-1.155 |
| **Hilar clamping**, minutes, ≥30 | 0.296 | 0.792 | 0.511-1.227 | 0.769 | 0.938 | 0.614-1.434 |
| **Gross type**, no mass-forming | 0.782 | 0.892 | 0.396-2.007 | 0.062 | 0.266 | 0.066-1.071 |
| **Cirrhosis**, yes | 0.195 | 1.413 | 0.837-2.384 | 0.797 | 0.916 | 0.470-1.787 |
| **Tumour size**, cm, ≥5 | 0.014 | 1.403 | 1.070-1.838 | 0.023 | 1.387 | 1.046-1.840 |
| **Tumour number**, multiple | <0.001 | 1.682 | 1.286-2.199 | <0.001 | 1.903 | 1.432-2.528 |
| **Adjacent organs invasion**, yes | <0.001 | 3.276 | 2.196-4.915 | <0.001 | 2.605 | 1.572-4.317 |
| **Lymph node metastasis**, yes | <0.001 | 1.985 | 1.507-2.614 | 0.005 | 1.545 | 1.138-2.098 |
| **Vascular invasion**, yes | <0.001 | 1.752 | 1.301-2.360 | 0.036 | 1.423 | 1.023-1.979 |
| **Differentiation**, moderate/well | 0.451 | 1.366 | 0.607-3.071 | 0.281 | 1.629 | 0.671-3.956 |
| **TNM**, III/IV | 0.001 | 1.549 | 1.184-2.027 | 0.171 | 1.228 | 0.915-1.649 |
| **Abbreviation**: ICC, intrahepatic cholangiocarcinoma; HBV, hepatitis B virus; OS, overall survival; HR, hazard ratio; CI, confidence interval; HCV, hepatitis C virus; TBIL, total bilirubin; ALB, Albumin; ALT, alanine aminotransferase; PT, prothrombin time; AFP, a-fetoprotein; CEA, carcinoembryonic antigen; CA 19-9, carbohydrate antigen 19-9; NLR, neutrophil to lymphocyte ratio; PLR, Platelet-Lymphocyte Ratio; PNI, prognostic nutritional index; TNM, tumour node metastasis. | | | | | | |
