# Supplementary material for: Elevated serum neutrophil-lymphocyte ratio is associated with worse long-term survival in patients with HBV-related intrahepatic cholangiocarcinoma undergoing resection
Source: Front Oncol. 2022 Oct 17;12:1012246. doi: 10.3389/fonc.2022.1012246 (PMC9618718; doi:10.3389/fonc.2022.1012246)
Supplement: Supplementary file 4 [file Table_4.docx]

| **Supplemental Table 4. Univariate analysis of prognostic factors in ICC patients with HBV infection in the PSM cohort** | | | | | | |
| --- | --- | --- | --- | --- | --- | --- |
| **Variable** | **OS** | | | **Tumour recurrence** | | |
|  | ***P*-value** | **HR** | **95%CI** | ***P*-value** | **HR** | **95%CI** |
| **Age**, years, >60 | 0.866 | 1.027 | 0.752-1.403 | 0.435 | 0.887 | 0.656-1.199 |
| **Sex**, male | 0.792 | 0.963 | 0.729-1.272 | 0.749 | 0.958 | 0.734-1.249 |
| **Hepatolithiasis**, yes | 0.325 | 1.222 | 0.820-1.820 | 0.280 | 0.789 | 0.512-1.214 |
| **Anti-HCV**, positive | 0.142 | 2.358 | 0.750-7.414 | 0.709 | 1.305 | 0.323-5.280 |
| **TBIL**, µmol/L, >17 | 0.091 | 0.739 | 0.520-1.050 | 0.348 | 0.856 | 0.620-1.184 |
| **ALB**, g/L, ≥35 | 0.612 | 0.833 | 0.411-1.689 | 0.811 | 1.096 | 0.517-2.327 |
| **ALT**, U/L, >80 | 0.895 | 1.037 | 0.601-1.791 | 0.688 | 1.114 | 0.657-1.889 |
| **PT**, seconds, >13 | 0.027 | 1.706 | 1.063-2.736 | 0.017 | 1.747 | 1.104-2.765 |
| **AFP**, µg/L, >20 | 0.183 | 1.241 | 0.903-1.705 | 0.032 | 1.385 | 1.028-1.867 |
| **CEA**, µg/L, >10 | <0.001 | 2.228 | 1.473-3.370 | 0.003 | 1.911 | 1.250-2.923 |
| **CA 19-9**, U/L, >39 | 0.001 | 1.554 | 1.185-2.038 | 0.034 | 1.323 | 1.021-1.714 |
| **NLR**, ≥2.15 | 0.013 | 1.414 | 1.077-1.857 | 0.010 | 1.410 | 1.087-1.830 |
| **PLR**, ≥141 | 0.307 | 1.199 | 1.846-1.699 | 0.994 | 0.987 | 0.694-1.405 |
| **PNI**, ≥46.5 | 0.454 | 0.868 | 0.598-1.258 | 0.733 | 1.067 | 0.734-1.551 |
| **Operation time**, hours, ≥3 | 0.894 | 0.974 | 0.662-1.434 | 0.247 | 0.797 | 0.543-1.170 |
| **Hilar clamping**, minutes, ≥30 | 0.324 | 1.249 | 0.803-1.943 | 0.237 | 1.291 | 0.846-1.972 |
| **Gross type**, no mass-forming | 0.773 | 1.157 | 0.430-3.115 | 0.722 | 0.813 | 0.260-2.544 |
| **Cirrhosis**, yes | 0.274 | 1.174 | 0.880-1.568 | 0.084 | 1.272 | 0.968-1.670 |
| **Tumour size**, cm, ≥5 | <0.001 | 1.776 | 1.341-2.353 | <0.001 | 1.903 | 1.453-2.492 |
| **Tumour number**, multiple | <0.001 | 1.741 | 1.289-2.350 | <0.001 | 1.937 | 1.455-2.578 |
| **Adjacent organs invasion**, yes | <0.001 | 2.521 | 1.552-4.093 | 0.002 | 2.214 | 1.330-3.685 |
| **Lymph node metastasis**, yes | 0.004 | 1.697 | 1.185-2.432 | 0.005 | 1.673 | 1.171-2.389 |
| **Vascular invasion**, yes | <0.001 | 2.099 | 1.552-2.840 | <0.001 | 2.203 | 1.640-2.960 |
| **Differentiation**, moderate/well | 0.048 | 0.567 | 0.323-0.996 | 0.467 | 0.798 | 0.435-1.464 |
| **TNM**, III/IV | 0.002 | 1.673 | 1.212-2.309 | 0.003 | 1.625 | 1.182-2.234 |
| **Abbreviation**: ICC, intrahepatic cholangiocarcinoma; HBV, hepatitis B virus; PSM, propensity score matching; OS, overall survival; HR, hazard ratio; CI, confidence interval; HCV, hepatitis C virus; TBIL, total bilirubin; ALB, Albumin; ALT, alanine aminotransferase; PT, prothrombin time; AFP, a-fetoprotein; CEA, carcinoembryonic antigen; CA 19-9, carbohydrate antigen 19-9; NLR, neutrophil to lymphocyte ratio; PLR, Platelet-Lymphocyte Ratio; PNI, prognostic nutritional index; TNM, tumour node metastasis. | | | | | | |
