# Supplementary figures and images for: Elevated serum neutrophil-lymphocyte ratio is associated with worse long-term survival in patients with HBV-related intrahepatic cholangiocarcinoma undergoing resection
Source: Front Oncol. 2022 Oct 17;12:1012246. doi: 10.3389/fonc.2022.1012246 (PMC9618718; doi:10.3389/fonc.2022.1012246)

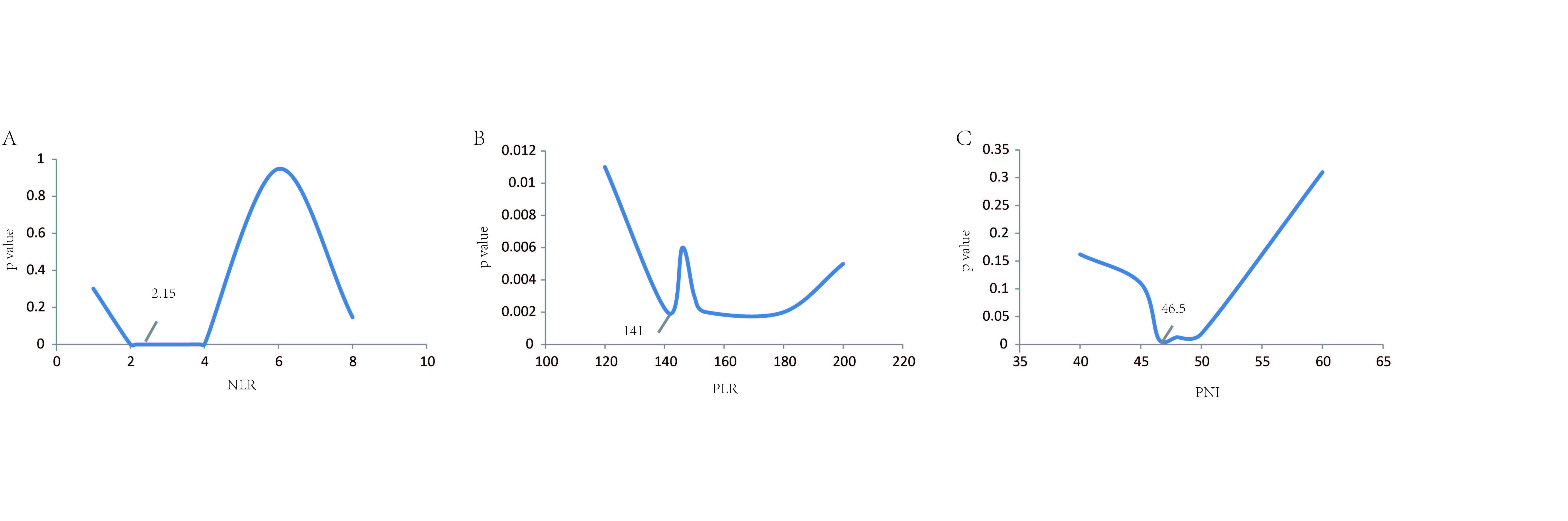

Supplement: Supplementary Figure 1 — Calculation process of the minimum p-value of the cut-off values of neutrophil-lymphocyte ratio, platelet-lymphocyte ratio, and prognostic nutritional index. [file Image_1.jpeg]
